# PLCE1 is a poor prognostic marker and may promote immune escape from osteosarcoma by the CD70-CD27 signaling pathway

**DOI:** 10.17305/bjbms.2022.7416

**Published:** 2022-06-26

**Authors:** Linhai Huang, Chundi Liao, Hanhua Wu, Piwei Huang

**Affiliations:** 1 Division of Orthopedic Surgery, Wuming Hospital of Guangxi Medical University, Wuming District, Nanning, China; 2 Division of Radiology, Wuming Hospital of Guangxi Medical University, Wuming District, Nanning, China; 3 Division of Spinal Surgery, The First Affiliated Hospital of Guangxi Medical University, Nanning, China

**Keywords:** PLCE1, osteosarcoma, tumor immunity, tumor microenvironment, tumor immune escape

## Abstract

Phospholipase C epsilon 1 (PLCE1) is involved in the pathogenesis of many cancers. However, the biological role of PLCE1 in osteosarcoma (OS) is still poorly understood. The prognostic survival analysis was performed on the *PLCE1* gene in the TARGET dataset and the differential expression of *PLCE1* in OS tissue and normal bone tissue on the tissue chip was detected by immunohistochemistry. Spearman’s rank correlation coefficient analysis was implemented to explore the relationship between *PLCE1* and immune genes. Finally, *PLCE1* was silenced to explore its biological function in OS cells. The results of tissue chip immunohistochemistry showed that *PLCE1* expression in OS tissue was higher than in normal bone tissue. The survival curve of *PLCE1* and its corresponding receiver operating characteristic curve (ROC) showed that PLCE1 had a significant effect on the survival status of patients with OS and that the prognosis of patients with high *PLCE1* expression was relatively poor. Spearman’s rank correlation coefficient analysis and qRT-PCR assays found that PLCE1 may promote immune escape from OS via CD70-CD27 signaling pathway. Silencing of *PLCE1* causes the following biological behaviors of OS cells: It promotes apoptosis, inhibits proliferation of OS cells, and inhibits the ability of cell migration and invasion. PLCE1 is a poor prognostic marker and a potential key factor affecting the immune status of the OS tumor microenvironment.

## INTRODUCTION

Osteosarcoma (OS) is more common in adolescents and is a common primary malignant bone tumor [1]. Due to the high degree of malignancy of OS, lung metastases often occur early in patients [2]. Although the clinical treatment of OS has made some progress recently, the overall survival rate of patients with OS has not been significantly improved [3]. Therefore, it is urgent to explore molecular mechanisms and find new therapeutic targets to provide more options for clinical diagnosis and treatment of OS. Studies have reported that the tumor microenvironment (TME) is considered to be the target of tumor drug targeted therapy in the future [4]. In addition to the currently used anti-PD-1/PD-L1 or anti-CTLA-4 therapeutic drugs, cancer-associated fibroblasts and tumor-associated macrophages (TAMs) in the microenvironment are also considered as potential targets for cancer treatment [5, 6]. These treatment strategies provide new ideas for the treatment of OS.

Phospholipase C epsilon 1 (PLCE1) belongs to the phosphoinositide-specific phospholipase C family [7]. The production of the second messenger molecules such as inositol 1,4,5-trisphosphate and diacylglycerol is regulated by activated phosphatidylinositol-specific phospholipase C enzymes [8]. PLCE1, which mediates small GTPases of the Ras superfamily through the activity of its Ras guanine exchange factor, is a bifunctional enzyme [7]. As the effector of heterotrimer and small G protein, it is involved in regulating cell growth, cell survival, T-cell activation, and actin organization [9]. Studies have revealed that mutations in the *PLCE1* gene or changes in *PLCE1* expression are closely related to the susceptibility to a variety of tumors, such as gastric [10], colorectal [11], and renal cancer [12]. Hydroxymethylation-related activation of PLCE1 prevents autophagy and promotes esophageal carcinogenesis through MDM2-mediated ubiquitination and p53 instability [13]. Moreover, in podocytes, PLCE1 regulates the formation of lamellar lipoproteins and acts downstream of the actin-binding protein advillin to allow the assembly of the ARP2/3 complex [14]. Despite these findings, the clinical significance and biological function of PLCE1 in OS are still unknown. Therefore, we investigated the biological functions of PLCE1 in OS and studied its potential molecular mechanisms.

## MATERIALS AND METHODS

### Tissue chip immunohistochemistry

The tissue chip was purchased from Xi’an Taibosi Biological Co., Ltd. It contained 41 cases of OS, plus 9 normal tissues, each with a single core. The sections were carefully dewaxed with xylene and then hydrated with different concentration gradients of alcohol. The slices were soaked in a 3% H_2_O_2_ solution to remove endogenous catalase and then cooked with citric acid to repair the antigen. The non-specific sites were blocked by serum. Next, the primary antibody was added and incubated overnight in a refrigerator at 4°C, and then, the secondary antibody was incubated at 37°C for half an hour. Finally, the slices were scanned and analyzed.

### Survival analysis and Spearman rank correlation coefficient analysis of PLCE1

The data from OS tissue chip and their corresponding clinical information were collected for further analysis of the clinical significance of PLCE1 in OS.

The raw count of OS microarray data from 87 OS patients and their corresponding clinical information were obtained from the TARGET database of the National Institutes of Health (https://ocg.cancer.gov/programs/target). The patients were divided into two groups according to the *PLCE1* gene expression value, and Kaplan–Meier was implemented to analyze the survival differences between the two groups. Receiver operating characteristic (ROC) analysis at different times was performed to compare the predictive accuracy and risk score of PLCE1. Moreover, survival analysis on *PLCE1*-related genes was carried out. Visual analysis of protein-protein interaction networks was performed on related genes. R software version v4.0.3 was used for the abovementioned analysis methods.

### Association between *PLCE1* and *TME*

Studies have found that TME can affect the effect of immune checkpoint inhibitors treatment, and further exploration of the relationship between *PLCE1* and TME can provide clinical reference for treatment. Spearman correlation was performed to analyze the relationship between PLCE1 and immune checkpoints or TAM markers. The median of *PLCE1* was the cutoff point for dividing the high- and the low-expression group, and the differences of biomarkers were compared between the two groups.

### Immune characteristics of different *PLCE1* subgroups

The raw count of OS RNA-sequencing data from 98 OS patients and their corresponding clinical information were obtained from the TARGET database of the National Institutes of Health (https://ocg.cancer.gov/programs/target). The online website CIBERSORT (https://cibersort.stanford.edu/) was used to identify the immune characteristics of OS. The expression matrix data were imported online and repeated 1000 times. The median of *PLCE1* was the cutoff point for dividing the high- and low-expression group. We then calculated and compared the differences of 22 types of immune cells between the two *PLCE1* subgroups.

### Cell cultures

McCoy’s 5a (Gibco, US) was used to cultivate human OS U-2OS and Saos2 cells, RPMI1640 (Gibco, US) was used to cultivate human OS MG-63 and HOS cells, and DMEM/F12 (Gibco, US) was used to cultivate hFOB1.19 cells. The medium contained 10% fetal bovine serum (FBS, Gibco, US) and 1% penicillin-streptomycin (PS, Gibco, US). The cells were cultured in an incubator containing 5% CO_2_ at 37°C. The above cell lines were obtained from the Cell Bank of the Chinese Academy of Sciences.

### Cell transfection and quantitative real-time PCR (RT-PCR) assays

The siRNAs (Generay, China) were separately transfected into the cells with Lipofectamine 3000 (Invitrogen, USA). The cells were then grown for 6 hours, after which media were exchanged for fresh complete media and then further incubated for 48 hours. TRIzol (Gibco, US) was used to extract total cell RNA following the reagent instructions, and then, RT-PCR was performed. The results were analyzed by the 2^−DDCt^ method. Three siRNAs (siRNA1-sense: 5’-GGUUGUUCAUUAAGAGUAATT-3’, siRNA1-antisense: 5’-UUACUCUUAAUGAACAACCTT-3’; siRNA2-sense: 5’- GA UGUUUAAUACAGAAGAATT-3’, siRNA2-antisense: 5’-UUCUUCUGUAUUAA ACAUCTT-3’; siRNA3-sense: 5’- GGAUAAAGAAAGCAGAUAATT-3’, siRNA3-antisense: 5’- UUAUCUGCUUUCUUUAUCCTT-3’) were synthesized from Shanghai Generay Biotech Co., Ltd. (Shanghai, China). Primers (Sangon Biotech Company, China) were as follows: PLCE1-hF GCTTCTTAACACGGGACTTGG, PLCE1-hRCTTCAAGGGCATTGTGCTCTC; CD70-hFGCT T T GGTCCCATTGGT CG, CD70-hR CGTCCCAC CC AAGTGACTC; and GAPDH-hFTGACAACTTTG GTATC GTGGAAGG, GAPDH-hRAGGCAGGGATGATGTT CGGAGAG. The study groups used in this research are as follows: Blank, si-NC, si-PLCE1-1, si-PLCE1-2, and si-PLCE1-3.

### Western blotting

RIPA lysis buffer (Beyotime Biotechnology, China) was used to extract total cell protein. The sample was placed on ice for 30 minutes and then centrifuged at 12,000 g at 4°C for 10 minutes. BCA Protein Assay Kit (Thermo, US) was used to detect protein concentration. SDS-PAGE gel (Beyotime Biotechnology, China) was used to separate protein samples. The protein was first transferred to the PVDF membrane (Millipore, US), then blocked with 5% skimmed milk powder, and finally incubated with the primary antibody (Affinity, US) overnight at 4^o^C. The blot was incubated with the secondary antibody (Jackson ImmunoResearch, US) for 2 hours at 37°C and finally detected by ECL kit (Beyotime Biotechnology, China). Each experiment was repeated 3 times. Gray values were analyzed with ImageJ.

### Cell proliferation

CCK8 (Beyotime Biotechnology, China) was used to detect cell proliferation following the reagent instructions. Cells were seeded in 96-well plates at an appropriate density and then cultured at 37°C in a humidified incubator containing 5% CO_2_. Cells were then transfected and incubated for 48 hours. A 10 μl of CCK8 solution was added to each well and incubated for 1 hour. Finally, the absorbance was measured, and the cell viability curve drawn.

### Colony formation assays

After the cells were transfected with the siRNAs for 24 hours, 200 cells/well were added to the 6-well plate and cultured in an incubator at 37°C for 14 days. When obvious cell clones were visible to the naked eye, the cells were fixed with 4% paraformaldehyde (China National Pharmaceutical Group Corporation, China) for 10 minutes at 37°C and stained with crystal violet (Beyotime Biotechnology, China) for 10 minutes at 37°C. Finally, the Petri dish was rinsed with distilled water to remove excess crystal violet. After drying, a photo was taken for recording.

### Apoptosis and cell cycle assays

Annexin V-FITC/PI Apoptosis Detection Kit (BD, US) was used to detect apoptosis and cell cycle following the manufacturer’s instructions. Cells were collected by centrifugation and resuspended in 1×binding buffer after being transfected for 48 hours. They were then incubated with Annexin V-FITC/PI in a dark room at 37°C for a quarter of an hour. Finally, the cells were mixed with 1×binding buffer. The apoptosis assays were detected by the flow cytometry FACSCalibur (BD, US) within one hour. Only cells that stained positive for Annexin V-FITC were considered apoptotic cells. The method used to quantify apoptosis was to calculate the ratio of the number of apoptotic cells to the total number of cells. For cell cycle assays, the cells were mixed with 70% ethanol pre-cooled at −20°C and fixed at 4°C overnight. After centrifugation, the cells were incubated with PBS containing RNase A at 37°C for 30 minutes and then mixed with propidium iodide. The cell cycle assays were detected by flow cytometry FACSCalibur (BD, US) within 24 hours after staining for 30 minutes in the dark. The stained cells were analyzed using FACSComp (BD, USA) software.

### Transwell assays

After resuspension in serum-free medium Opti-MEM (Gibco, US), cells in the upper chambers were incubated with matrix gel for invasion assays and without matrix gel for migration assays, while the lower chambers were supplemented with serum-containing medium MEM (Gibco, US). After 48 hours, the cells were fixed with 4% paraformaldehyde for 20 minutes at 37°C. Then, the cells in the upper chamber were wiped clean and the number of invading cells in the lower chamber was observed under a microscope and a photo taken for storage.

### Ethical statement

This study was done in accordance with the ethical principles of the 1964 Declaration of Helsinki. All research protocols were approved by the Human Experiment Committee of Wuming Hospital Affiliated to Guangxi Medical University (No: 2021(Z005)).

### Statistical analysis

Statistical analysis was conducted by GraphPad Prism 9 and SPSS23.0. The Kaplan–Meier analysis was implemented to analyze the prognostic survival of patients with OS. The accuracy of the survival curve was judged based on the area under the ROC. The t-test or the one-way variance was used to analyze the experimental data. Wilcoxon test and Spearman’s rank correlation were used in the study. All experiments were repeated at least 3 times, and *p*<0.05 was considered statistically significant.

## RESULTS

### *PLCE1* expression in OS

**FIGURE 1:**
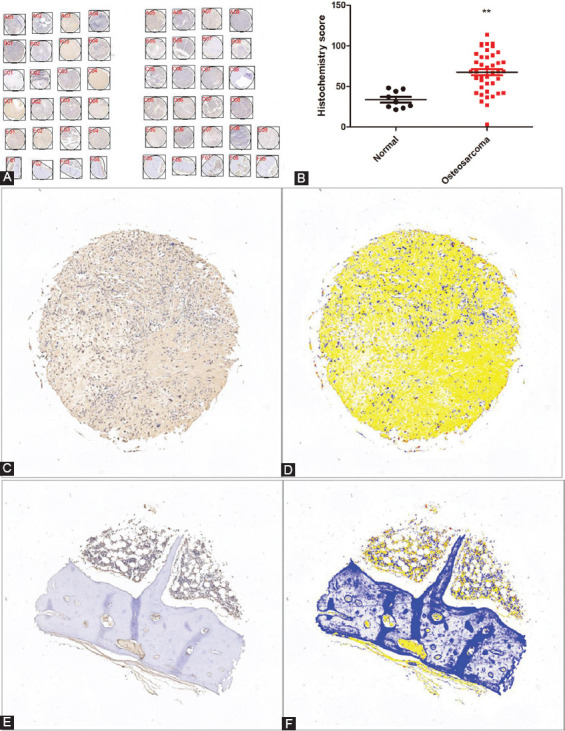
Expression of *PLCE1* in OS tissue microarray. (A) General view of immunohistochemical results of OS tissue chip (F01–F09 are normal bone tissues and A01–E09 are OS tissues). (B) The image analysis system analyzes the intensity of the immunohistochemical positive area (**p<0.01). (C and D) Immunohistochemical results of OS tissue and the corresponding image system analysis display (B–immunohistochemical stains; C–derived from system analysis display). (E and F) Immunohistochemical results of normal bone tissue and the corresponding image system analysis display (D–immunohistochemical stains; E–derived from system analysis display). PLCE1: Phospholipase C epsilon 1; OS: Osteosarcoma.

Immunohistochemical analysis was performed on the OS tissue chip to evaluate the expression of *PLCE1* in OS. It was found that PLCE1 was expressed in the cytoplasm. The histochemical score was performed on the results of tissue chip immunohistochemistry, and it was concluded that *PLCE1* was highly expressed in OS tissues (Figure 1A-F and Supplemental Figure 1).

### Expression and clinical characteristics of *PLCE1*

To investigate the value of *PLCE1* in OS, data from 41 OS cases from the tissue chip and their clinical characteristics were analyzed (Table 1). A significant difference in *PLCE1* expression levels was observed between tumor stages (*p*=0.037), with higher *PLCE1* expression in higher stages. A significant difference was also observed between metastatic and non-metastatic groups (*p*=0.021). There was no statistical significance between sex and age groups.

**TABLE 1:**
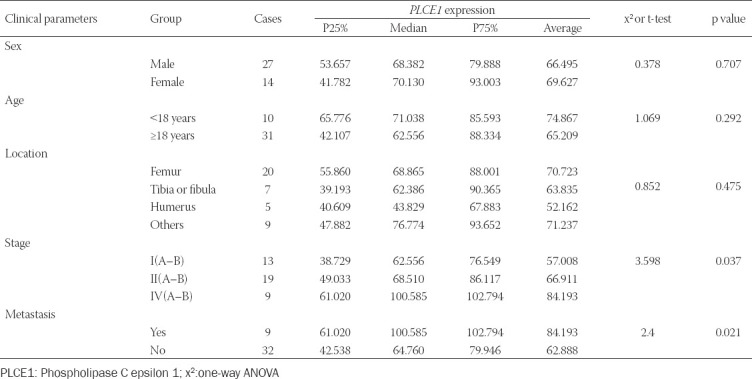
Relationship between *PLCE1* expression and clinical parameters

### Survival analysis of *PLCE1* gene expression

**FIGURE 2:**
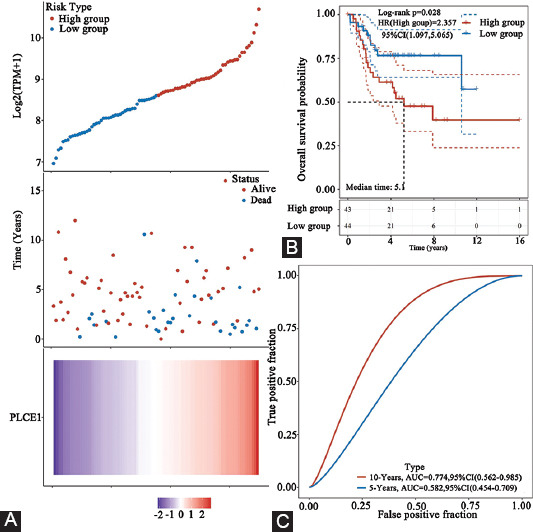
Survival analysis of *PLCE1* gene expression. (A) The upper figure represents the risk scoring curve; the middle figure represents the distribution of the survival time and survival status corresponding to the *PLCE1* expression; the lower figure represents the *PLCE1* expression heat map. The X-axis labels are the same for all panels, and the X-axis represents samples ordered by z-score of *PLCE1* expression. (B) Kaplan–Meier survival analysis of *PLCE1* gene (*p*=0.028). (C) Time-dependent ROC analysis of *PLCE1* gene. PLCE1: Phospholipase C epsilon 1.

In order to explore whether *PLCE1* is related to the prognosis of patients with OS, Kaplan–Meier survival analysis was used to analyze the TARGET database. The results showed that there was a significant difference in overall survival between the high-expression and the low-expression group (*p*<0.05, HR [high group] = 2.727). The standard of the risk model is verified by the ROC, and it was found that the model had a high accuracy rate (5 years, AUC=0.586; 10 years, AUC=0.774). The survival curve of *PLCE1* and its corresponding ROC is an integral part of the risk model, indicating that it has an important influence on the prognosis and survival status of patients with OS. The results indicate that the prognosis of patients with high PLCE1 expression is poor (Figure 2A-C).

### *PLCE1* and TME

**FIGURE 3:**
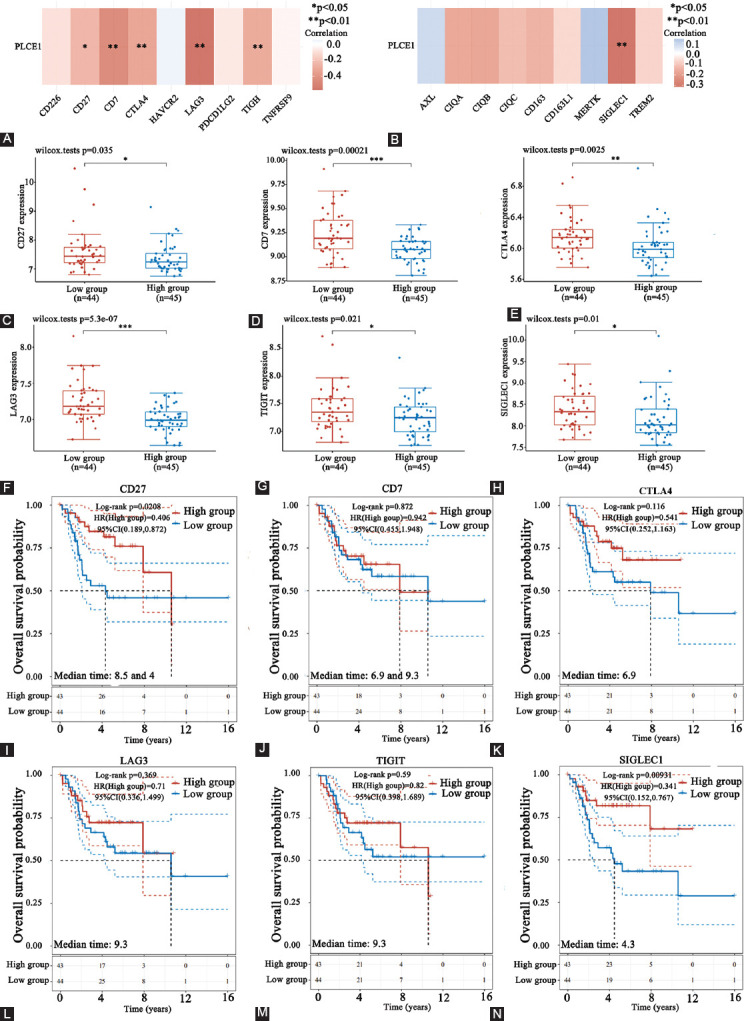
*PLCE1* and immune microenvironment. (A and B) Spearman’s rank correlation coefficient analysis of *PLCE1* and immune-related genes. (C-H) The expression of immune-related genes in *PLCE1* high and low groups. (I-N) Kaplan–Meier survival analysis of *PLCE1-*related genes (*p<0.05, **p<0.01). PLCE1: Phospholipase C epsilon 1.

In order to evaluate the impact of *PLCE1* on TME, Spearman’s rank correlation coefficient was used to analyze the relationship between *PLCE1* and immune checkpoints or TAM markers. In this study, it was found that *PLCE1* was negatively correlated with immune checkpoints CD27, CD7, CTLA4, LAG3, TIGIT, and TAMs marker SIGLEC1 (Figure 3A and B). The expression of these genes decreased in the *PLCE1* high-expression group (Figure 3C-H). We further explored the prognostic role of *PLCE1*-related immune genes in patients with OS. It was found that CD27 and SIGLEC1were positively correlated with the prognosis of patients with OS. Patients with high expression of CD27 and SIGLEC1 had better prognosis (Figure 3I-N). To explore the relationship between *PLCE1* and immune checkpoint CD27, the expression of *PLCE1* was knocked down to check the expression changes of its ligand CD70. After knocking down *PLCE1* expression in MG63 cells, it was found that CD70 expression also decreased (Figure 5C).

### Immune characteristics of different *PLCE1* subgroups

**FIGURE 4:**
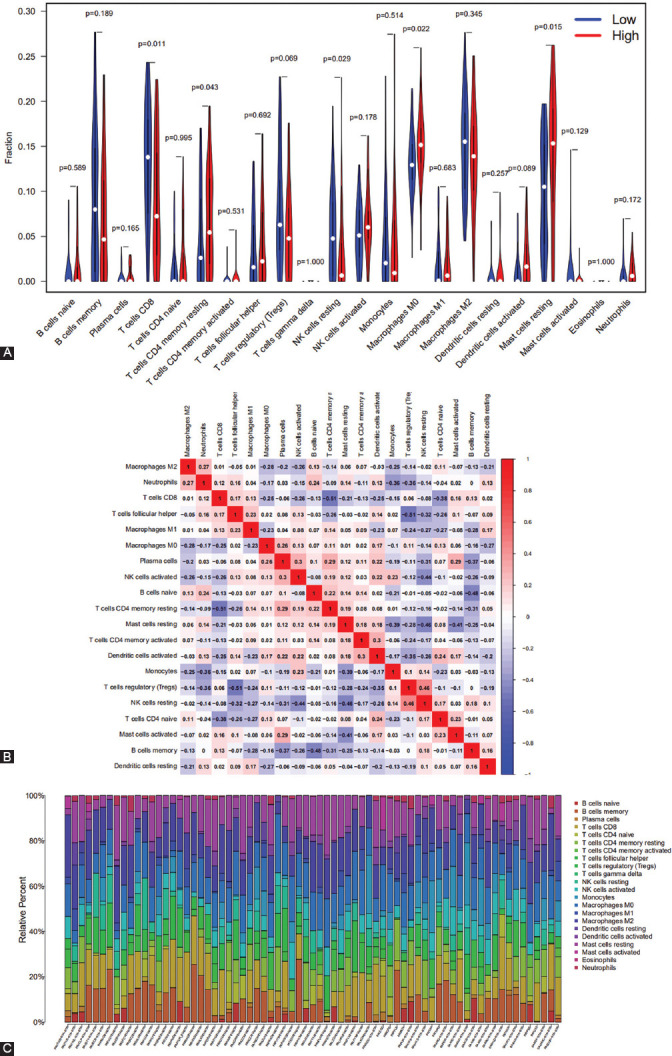
Immune characteristics of different *PLCE1* subgroups. (A) The composition and distribution of immune cells in different *PLCE1* subgroups. (B and C) The features relating to the immune landscape are shown in the figure. *PLCE1*: Phospholipase C epsilon 1; NK: Natural killer.

Wilcoxon test was performed to analyze the composition and distribution of immune cells in different *PLCE1* subgroups. It was found that resting memory CD4+ T cells, resting NK cells, M0 macrophages, and resting mast cells were more abundant in the high *PLCE1* subgroup, while CD8+ T cells were significantly more abundant in the low *PLCE1* subgroup (Figure 4A, *p*=0.011). Compared with the high *PLCE1* subgroup, the expression of regulatory T cells (Tregs) in the low *PLCE1* subgroup was increased, but not significant (Figure 4A, *p*=0.069). Furthermore, it was also found that activated dendritic cells were more abundant in the high *PLCE1* subgroup, but not significantly (Figure 4A, *p*=0.089). Features related to the immune landscape are shown in Figure 4B and C.

### Effect of *PLCE1* on OS cell proliferation

**FIGURE 5:**
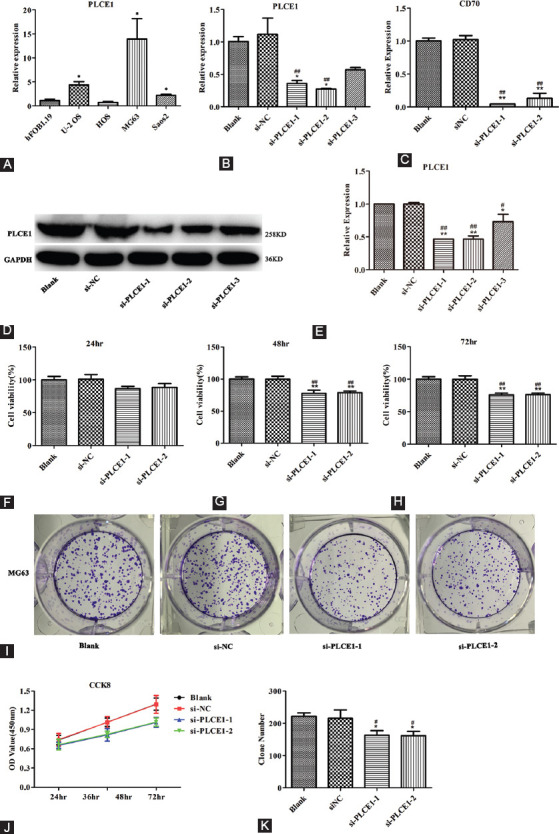
The effect of *PLCE1* knockdown on the proliferation and clone formation of OS cells. (A) Detection of the expression of *PLCE1* in hFOB1.19 and OS cell lines by qRT-PCR. (*means compared with hFOB1.19, *p<0.05) (B) Detecting the silencing efficiency of *PLCE1* with qRT-PCR (C) In MG63 cells, CD70 decreased with the silencing of *PLCE1*. (D and E) Detecting the silencing efficiency of *PLCE1* in MG63 cells with WB. (F-I) CCK8 analyzes the effect of silencing *PLCE1* on the proliferation of MG63 cell line. (J and K) The effect of silencing *PLCE1* on cell clone formation of MG63 cell line (* or ** means compared with blank group, # or ## means compared with si-NC group. *p<0.05, **p<0.01, ^#^*p*<0.05, and ^##^*p*<0.01). *PLCE1*: Phospholipase C epsilon 1; OS: Osteosarcoma; WB: Western blot.

qRT-PCR was used to detect the mRNA levels of *PLCE1* in four OS cell lines (Figure 5A). To study the biological function of *PLCE1* in OS cells, we introduced siRNA targeting *PLCE1* into MG63 cells for the next experiment. The results showed that the knockdown effect of siRNA-1 and siRNA-2 was more significant (Figure 5B-E). The CCK8 experiment was used to observe the effect of the knockdown of *PLCE1* on the proliferation of MG63 cells (Figure 5F-I). The results of the CCK-8 experiment showed that after knocking down *PLCE1* expression, the activity of MG-63 cells was reduced, and there were extremely significant differences at 48 hours and 72 hours, so 48 hours were selected for subsequent experiments. The results also revealed that knocking down *PLCE1* can significantly inhibit the cell clone formation of MG63 cells (Figure 5J-K).

### Effect of *PLCE1* on OS cell cycle and apoptosis

**FIGURE 6:**
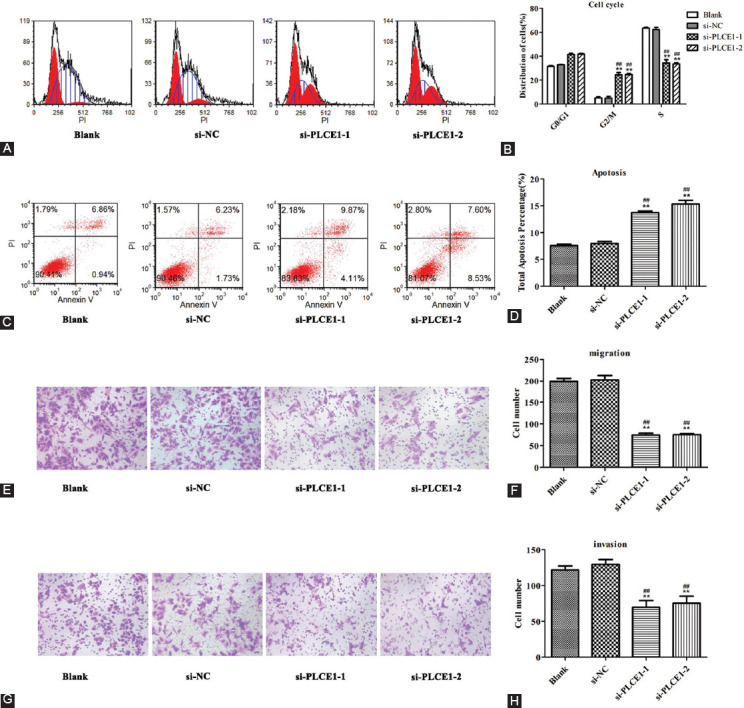
Effect of *PLCE1* on cell cycle, apoptosis, migration, and invasion. (A-D) Flow cytometry analysis and apoptosis assays showed that silencing *PLCE1* can prevent the cell cycle and inhibit cell apoptosis. (E and F) Migration assays showed that silencing *PLCE1* inhibits the migration ability of the MG63 cell line. (G and H) Invasion assays showed that silencing *PLCE1* inhibits the invasion ability of MG63 cell line (* or ** means compared with blank group, # or ## means compared with si-NC group. *p<0.05, **p<0.01, ^#^*p*<0.05, and ^##^*p*<0.01).

Flow cytometry analysis was performed to detect cell cycle distribution and apoptosis (Figure 6A-H). After knocking down *PLCE1*, the G2/M phase cycle ratio of MG63 cells was significantly increased and S phase cycle ratio of MG63 cells was significantly decreased, while the G0/G1 phase remained unchanged compared with blank and si-NC groups (Figure 6A and B, *p<0.05, **p<0.01, ^#^*p*<0.05, and ^##^*p*<0.01). In addition, the cell apoptosis assays showed that knocking down *PLCE1* significantly increased the apoptotic level of MG63 cells compared with blank and si-NC groups (Figure 6C and D, *p<0.05, **p<0.01, ^#^*p*<0.05, and ^##^*p*<0.01).

### Effect of *PLCE1* on OS cell migration and invasion

A transwell experiment was implemented to study the effect of *PLCE1* on the invasion and the migration of OS cells. The results showed that the number of invasions and migrations of MG63 cells was significantly reduced after *PLCE1* silencing compared with blank and si-NC groups (Figure 6E-H, *p<0.05, **p<0.01, #*p*<0.05, and ##*p*<0.01).

## DISCUSSION

OS is a highly malignant and aggressive bone tumor that often occurs in adolescents [1]. Current standard treatments include neoadjuvant chemotherapy, extensive surgical resection of the primary tumor, and recommended radiotherapy for patients who cannot be treated surgically [14]. Although some targeted drugs have made some progress in the treatment of OS in recent years, the 5-year overall survival rate is <70% [15]. The recurrence and/or metastasis rate of OS is still higher than 30% [16]. Due to resistance to chemotherapy or radiotherapy, the 5-year overall survival rate of some patients with recurrence and/or metastasis is <25% [17]. In recent years, immune checkpoint inhibitors have made great breakthroughs in the immunotherapy of various malignant solid tumors. However, anti-PD-L1 therapy has only a limited therapeutic effect on OS [4, 18]. Therefore, it is urgent to understand the molecular mechanism of the development of OS and determine more effective drug treatment targets. Our research found that *PLCE1* is highly expressed in patients with OS and affects the prognosis of patients. The expression of *PLCE1* is closely related to immune cells in TME.

Studies have found that PLCE1 regulates the migration, proliferation, and differentiation of podocytes [9]. Mutations in *PLCE1* can induce isolated diffuse mesangial sclerosis and increase the incidence of steroid-resistant nephrotic syndrome [19, 20], and *PLCE1* genetic defects lead to congenital nephrotic syndrome [21]. Many studies have focused on the relationship between single-nucleotide polymorphism variants of *PLCE1* and tumors, especially tumors of the digestive tract. The genetic variant *PLCE1* rs2274223 is significantly related to cardia cancer, and rs17417407 is related to the incidence of esophageal squamous cell carcinoma in humans [22, 23]. Multiple mutations at the 10q23 locus in *PLCE1* are associated with the high incidence of gastric and esophageal squamous cell carcinoma [24]. Many studies have found that *PLCE1* gene polymorphism not only regulates cell growth, differentiation, apoptosis, and angiogenesis [25] but also increases the susceptibility to esophageal [26], gastric [27], colon cancer [11], and squamous cell carcinoma of the head and neck [28]. It also affects the prognostic survival of patients [29]. *PLCE1* rs3765524 is associated with the susceptibility to dengue shock syndrome and may be the pathogenesis of severe dengue fever complications [30]. The hypomethylation of *PLCE1* can not only activate and hinder autophagy but also promotes tumorigenesis by ubiquitinating and destabilizing p53 mediated by MDM2 [13]. Hypomethylated *PLCE1* stimulates esophageal carcinoma angiogenesis and proliferation by activating the PI-PLC epsilon-NF-B signaling pathway and VEGF-C/Bcl-2 expression [31]. As a direct target of certain miRNAs, PLCE1 is involved in regulating the metastasis and growth of esophageal tumor and non-small cell lung cancer [32].

Although research on PLCE1 has made progress in other tumors, its role in OS has not been well characterized. Here, we found that the expression of *PLCE1* was elevated in the immunohistochemical staining of OS. Moreover, Kaplan–Meier survival analysis found that *PLCE1* is associated with poor patient prognosis. Patients with high *PLCE1* expression had a poor prognosis, and the overall survival rate was lower than that of the patients with low *PLCE1* expression. Therefore, it can be reckoned that *PLCE1* may serve as a potentially attractive new therapeutic target for OS. In addition, we conducted cell function experiments by silencing *PLCE1*. The results showed that after knocking down *PLCE1* expression, the ability of cell migration, proliferation, and invasion was weakened, the number of cell apoptosis increased, the cell G2/M phase increased, and the S phase decreased. Our results further support the role of high *PLCE1* expression in enhancing the proliferation of malignant cells and promoting tumor growth. In our research, the potential diagnostic capability of PLCE1 was determined and verified. Therefore, PLCE1 is expected to become a target for the diagnosis and treatment of OS.

At present, there are still many difficulties in the surgical treatment of OS, especially metastatic OS. Although a small number of cancer patients have benefited from anti-PD-1 antibody therapy, they still face major problems that need to be resolved, such as inefficiency and drug resistance [18]. Recent studies have shown that cancer cells and tumor-infiltrating cells are also involved in drug resistance. However, little is known about these underlying mechanisms. Therefore, the intratumoral heterogeneity of cancer has attracted more and more attention from researchers. Extensive heterogeneity is one of the important characteristics of tumors, which may lead to different responses of patients to the same treatment. Although many efforts have been made to clarify the heterogeneity of tumors, the current understanding of it is still mainly limited to tumor cells. TME is a cellular environment that includes immune cells, endothelial cells, mesenchymal cells, extracellular matrix molecules, and inflammatory mediators. The bone microenvironment of OS is composed of osteoclasts, osteoblasts, and hematopoietic cells, which are derived from monocytes/macrophages [33]. A variety of growth factors and cytokines released by these cells into the microenvironment play an important role in tumor development [34]. Therefore, a comprehensive analysis of the correlation between *PLCE1* and TME can provide a reference for the pathogenesis of OS.

In this study, we used Spearman’s rank correlation coefficient analysis to explore the correlation between *PLCE1* and TME, which revealed that *PLCE1* is involved in regulating immune cells in the microenvironment. It was found that *PLCE1* is closely related to immune checkpoint CD27 and TAM marker SIGLEC1. Recent studies have shown that the function of PLCE1 is involved in T-cell adhesion [35]. T-cell adhesion is conducive to the body’s routine immune surveillance and promotes the inflammatory reactions. Small GTPase Ras-proximate-1 or Ras-related protein 1 (Rap1) and its effectors play an important role in T-cell adhesion and integrin activation [7]. Rap1 is activated when combined with guanine exchange factor and exists in the form of GTP, while it is inactivated when combined with GTPase activator protein and exists in the form of GDP. Studies have found that PLCE1 has dual enzymatic function–it can digest fat and activate guanine exchange factor [36]. Studies have found that PLCE1 induces T-cell adhesion by activating the stromal cell-derived factor 1α/C-X-C chemokine receptor type 4 signal axis [7]. Moreover, it was found that PLCE1 induces lymphocyte adhesion and migration to inflammation sites by regulating SDF-1α [7] and suppresses tumor growth by regulating the mobilization of mouse T cells [35].

The prognostic value of *PLCE1*-related immune genes was evaluated in patients with OS, and immune checkpoint CD27 and TAM marker SIGLEC1 were found to be positively correlated with the prognosis of patients. The level of *PLCE1* expression is closely related to the degree of immune cell infiltration in TME. In this study, it was found that memory CD4+ T cells, NK cells, and mast cells in the *PLCE1* high-expression group were in a resting state, macrophages did not undergo M1 and M2 polarization, and CD8+ T cells were significantly more abundant in the *PLCE1* low-expression group. The above analysis found that PLCE1 regulated the microenvironment of OS.

SIGLEC1, also known as sialoadhesin or CD169, is a member of the immunoglobulin superfamily and is mainly expressed in macrophages, dendritic cells, and interferon-induced monocytes [37, 38]. Studies have shown that CD169+ macrophages in TME can inhibit the progression of a variety of malignant tumors [39, 40]. In TME, CD169 promotes cytotoxic T lymphocyte response, and the infiltration of CD169+ macrophages in the tumor enhances the cytotoxicity, proliferation, and cytokine production of CD8+ T cells [40]. It was found that the number of CD169+ macrophages in tumor tissues was significantly less than that in adjacent normal tissues, and patients with large numbers of CD169+ macrophages in tumor tissues had a better clinical prognosis [39, 40]. In this study, it was found that CD169 was decreased in the *PLCE1* high-expression group, and it was positively correlated with the patient’s clinical prognosis.

Tumor necrosis factor (TNF) is a multifunctional pro-inflammatory cytokine, mainly secreted by macrophages and combined with its receptors TNFRSF1A/TNFR1 and TNFRSF1B/TNFBR [41]. It regulates cell proliferation, differentiation, apoptosis, lipid metabolism, blood coagulation, and other biological processes [42, 43]. CD70, the ligand of TNFRSF7/CD27, belongs to the cytokine of TNF ligand family. It can activate the surface antigens of T and B lymphocytes, induce costimulatory T-cell proliferation, enhance the production of cytolytic T cells, and help T-cell activation [44]. It also plays a role in regulating the cytotoxic function of NK cells, B-cell activation, and immunoglobulin synthesis [45].

The CD27 protein is a member of the TNF receptor superfamily, which is necessary for the generation and long-term maintenance of T-cell immunity. It can regulate B-cell activation and immunoglobulin synthesis when combined with the ligand CD70 [46]. It has been found that CD70 is the only CD27 ligand, which is normally expressed transiently in highly activated T, NK, and B cells, and a small number of dendritic cells. However, it is highly expressed in various types of hematological malignancies [47], solid primary tumors [48], and metastatic tumors [49]. CD70 is an important immunomodulator that is immunosuppressive in TME and promotes immune escape [50]. The CD70-CD27 pathway enhances the malignant phenotype of diffuse malignant mesothelioma of the pleura and reduces the patient’s anti-tumor immune response [49]. In this study, it was found that after knocking down *PLCE1* expression in MG63 cells, CD70 expression also decreased. Interestingly, this result is in contrast to the previous data analysis, which may be due to data bias, a small sample size, or that CD70 is affected by other genes *in vivo*. Their expression is positively correlated in OS cells. In response to this phenomenon, we will further collect clinical specimens to detect their expression levels and conduct modeling in mice for further verification. Based on the above analysis, it was found that PLCE1 could regulate the microenvironment of OS through the CD70-CD27 pathway and mediate the immune escape of OS.

## CONCLUSION

In general, we confirmed that *PLCE1*, as a proto-oncogene, is highly expressed in OS and affects the prognostic survival of patients, as well as regulates the proliferation and invasion of OS. In addition, it was found that PLCE1 participates in the biological functions of OS by regulating TME. These findings provide new insights into PLCE1 as a potential diagnostic biomarker and effective molecular therapeutic target.
